# Generalized anxiety disorder patients' cognitive control in affective contexts

**DOI:** 10.3389/fpsyt.2025.1506239

**Published:** 2025-05-16

**Authors:** Yuqi Shen, Shasha Zhu, Shiqi Liao, Yuqing Zhao, Zihan Lin, Ke Jiang, Wenjing Yan, Xinhua Shen

**Affiliations:** ^1^ Huzhou Third Municipal Hospital, the Affiliated Hospital of Wenzhou Medical University, Huzhou, China; ^2^ School of Mental Health, Wenzhou Medical University, Wenzhou, China; ^3^ Department of Psychology, School of Mental Health, Wenzhou Medical University, Wenzhou, China; ^4^ Department of Neurosis and Psychosomatic Diseases, Huzhou Third Municipal Hospital, Huzhou, China

**Keywords:** generalized anxiety disorder, affective inhibition, affective shifting, cognitive control, affective control

## Abstract

**Background:**

Growing evidence suggests a relationship between deficits in cognitive control and anxiety. However, studies examining cognitive control within affective contexts (affective control) are limited, and the specific characteristics of affective control in patients with Generalized Anxiety Disorder (GAD) remain unclear. This study investigated whether differences exist in cognitive control under affective contexts.

**Methods:**

We conduct our research in a population of GAD patients (n = 50) and a healthy control group (n = 50). The affective flanker task measured affective inhibition, while the affective flexibility task assessed affective shifting capabilities.

**Results:**

GAD patients exhibited abnormal affective inhibition, characterized by reduced proactive control related to target stimulus processing and enhanced reactive control associated with distractor resolution. Additionally, GAD patients demonstrated deficits in affective shifting, as indicated by significantly higher shifting costs in both non-affective and affective tasks compared to the healthy control group.

**Conclusions:**

The findings reveal that GAD patients display poorer emotion recognition abilities, indicating deficits in affective control compared to healthy individuals. Our study underscores the importance of measuring affective control by delineating it into distinct components.

## 1 Introduction

Generalized anxiety disorder (GAD) is a prevalent chronic mental illness that often accompanies varying degrees of cognitive dysfunction, significantly impacting patients' quality of life and social functioning (1, 2). Research by Professor Phillips indicates that the monthly prevalence of anxiety disorders in China is 5.6%, with GAD accounting for a monthly prevalence of 1.3% (3). Anxiety represents an unpleasant emotional experience or psychological state that individuals encounter. As a fundamental emotion evolved during human history, moderate anxiety carries adaptive significance. However, high levels of anxiety distinctly impair cognitive abilities in individuals (4, 5). Some theoretical models propose that basic cognitive processing biases are underlying mechanisms for the development of anxiety disorders, including attentional bias to negative external stimuli, where anxious individuals detect negative information more quickly than neutral stimuli and amplify the threat level of the information, which can lead to hypervigilance or increased anxiety symptoms (6, 7).Goodwin et al. conducted a retrospective statistical analysis, the results of which indicated that in excess of 75% of the studies demonstrated a significant attentional bias for negative stimuli in the GAD group in comparison to healthy controls (8).

Cognitive control refers to conscious, top-down neurocognitive processes involving conscious, goal-directed control of thoughts, actions, and emotions (9). Miyake et al. revealed three core components of cognitive control through factor analyses: inhibition, shifting, and updating (10). Inhibition is an integrative system that involves controlling one's attention, behavior, thoughts, and emotions to override strong internal tendencies or external temptations in favor of what is more appropriate or needed (11). Both early theories of cognitive interference and processing efficacy, as well as more recent theories of attentional control, suggest that anxiety impairs an individual's inhibition control (4, 12). Shifting refers to the process by which individuals switch forward and backward between multiple tasks, operations, or mental fixations, rapidly switching from one task to another while completing various tasks (13). A persistent state of anxiety consumes a large amount of cognitive resources, making it difficult for individuals with GAD to effectively redistribute attention when attentional shifting or task switching is required, resulting in reduced cognitive efficiency (14).

Traditionally, measures of cognitive control do not include affective stimuli, but affective stimuli are more attention-grabbing or take up cognitive resources for people with GAD (15). In particular, difficulties in disengaging from unrelated affective stimuli and difficulties in focusing and processing new information may ultimately lead to maladaptive emotion regulation strategies such as avoidance (16). Indeed, linking cognitive control to affective stimuli has only recently led to a range of discussions. It is unclear whether cognitive control in individuals with GAD may be affected in the affective environment (17). Affective control refers to the application of the three components of cognitive control to the affective environment (18). According to attentional control theory, the core components of cognitive control, inhibition and shifting, require the involvement of attentional control functions, while updating requires the involvement of memory functions (4). Previous research has demonstrated that individuals with high anxiety have deficits in attentional control and are more susceptible to irrelevant information (19), and thus anxiety primarily impairs inhibition and shifting functions. In the present study, we focused on characterizing the inhibition and shifting functions of individuals with GAD in affective contexts.

Previous experimental designs for assessing inhibition have often used methods such as the Stroop task and the Flanker task. Previous studies have used cueing or internal switching tasks when assessing shifting ability. These experimental paradigms have two modes: one measuring cognitive control over neutral information and the other measuring cognitive control over affective information. Most previous studies of cognitive control have used neutral stimuli, and even when some studies have used affective stimuli, they have only used sketchy faces (20). However, facial emotion recognition is a specific cognitive domain that involves understanding the emotions of others based on their facial expressions. furthermore, the correct recognition of facial expressions is important for normal communication and social functioning. Patients with GAD report poor quality of life, impaired overall cognitive functioning, and social and interpersonal difficulties, which largely contribute to further exacerbating the degree to which anxiety disorders develop (21). The selection of sketchy faces in the study differed significantly from the patients’ affective recognition in their daily lives, and did not represent the patients’ affective control well. In addition, most studies on the relationship between affective control and anxiety have relied on college student samples with high trait anxiety populations, and have not extended the use of cognitive control in affective contexts to clinical GAD patients (17, 22, 23). Therefore, clarifying the characteristics of affective control in patients with GAD is necessary to help develop more effective and targeted interventions.

The current study aims to clarify the characteristics of GAD patients in different components of affective control compared with healthy participants. By analyzing the specific manifestations of affective inhibition and affective shifting function in GAD patients, we hope to reveal the relationship between abnormal affective control and GAD, and provide new perspectives and strategies for clinical treatment. We predicted that affective inhibition and affective shifting were associated with participants' self-reported mental health problems.

## 2 Method

### 2.1 Participants

**Figure 1:**
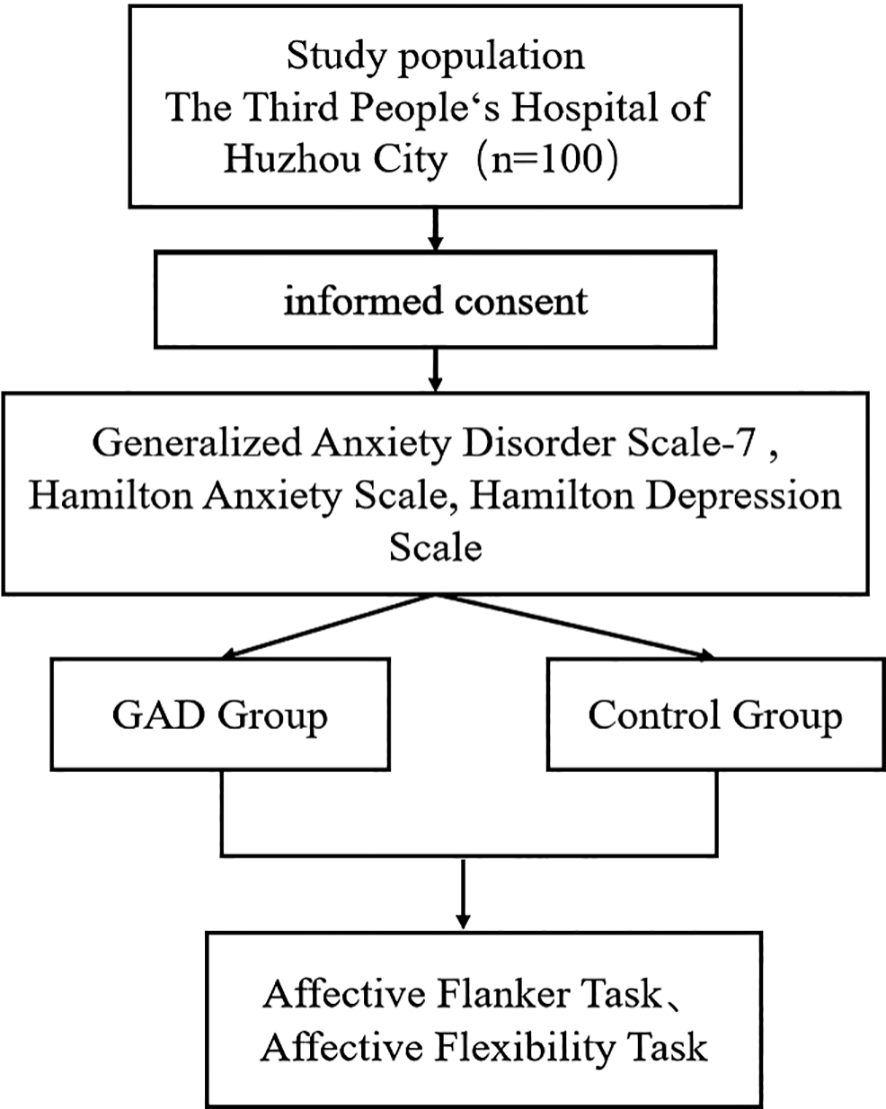
Flow chart for subject enrollment.

Using G*power 3.1 software (24), we calculated the sample size needed for the experiment. We set the effect size to a medium level of *ρ* = 0.25, *α* to 0.05, and statistical power to 0.80, determining that at least 32 participants are necessary. From 2023 to 2024, this study included 50 patients with GAD at the Third People's Hospital of Huzhou (**Figure 1**). Inclusion criteria were: (1) age between 18-60; (2) diagnosed with GAD by a licensed psychiatrist according to DSM-5 criteria; (3) Hamilton Anxiety Scale (HAMA) score ≥14 and Hamilton Depression Scale (HAMD-17) score <17; (4) at least a junior high school education; (5) ability to understand the study process; (6) right-handedness; (7) signed informed consent. Exclusion criteria included: (1) meeting DSM-5 criteria for other mental disorders; (2) presence of any physical or mental conditions that may affect task completion; (3) having undergone treatments like electroconvulsive therapy, transcranial magnetic stimulation, spinal cord, or deep brain stimulation in the past month. Concurrently, we recruited 50 healthy adults matched by gender and age, whose HAMA scores were < 7 and HAMD-17 scores were < 7, to form the control group (HC). This study received approval from the ethics review committee of the Third People's Hospital of Huzhou, and we obtained consent from all participants before enrollment, who signed informed consent forms.

### 2.2 Clinical assessment

#### 2.2.1 Generalized anxiety disorder scale-7

The GAD-7 is developed by the American Psychiatric Association based on DSM-IV diagnostic criteria for anxiety symptoms. It evaluates the frequency of anxiety symptoms experienced by participants over the past two weeks. The GAD-7 consists of seven items, using a four-level scoring system. Each item scores from "not at all, several days, more than half the days, nearly every day," corresponding to scores of "0, 1, 2, 3." The total score ranges from 0 to 21. The internal consistency reliability coefficient of the GAD-7 is 0.92, and the test-retest reliability is 0.83 (25). In this study, the Cronbach's α coefficient of GAD-7 is 0.894.

#### 2.2.2 Hamilton anxiety scale

The Hamilton Anxiety Rating Scale, developed by Hamilton in 1959, comprises 14 items. Clinically, this scale often serves as a basis for diagnosing anxiety disorders and classifying their severity. Each item on the HAMA uses a five-point rating system ranging from 0 to 4, with the following criteria: 0 indicates no symptoms; 1 indicates mild symptoms; 2 indicates moderate symptoms; 3 indicates severe symptoms; and 4 indicates very severe symptoms. HAMA demonstrates high reliability and validity, with an overall reliability coefficient r of 0.93. The reliability coefficients for individual symptom ratings range from 0.83 to 1.00, while the validity coefficient stands at 0.36 (26). In this study, HAMA's Cronbach's α coefficient is measured at 0.921.

#### 2.2.3 Hamilton depression scale

The Hamilton Depression Rating Scale, developed by Max Hamilton in 1960, serves as a common tool for assessing depressive states. This scale encompasses various versions, including 17, 21, and 24 items, with the 17-item version (HAMD-17) being the most frequently utilized. In this study, we employed the HAMD-17. This tool quantifies the severity of depression by evaluating the patient's symptoms. Each item typically utilizes a scoring method ranging from 0 to 4, where 0 indicates no symptoms and 4 reflects severe symptoms. The total score reliability coefficient r of the HAMD is 0.99, and the validity coefficient is 0.37 (27). In this study, HAMD's Cronbach's α coefficient is measured at 0.811.

### 2.3 Apparatus

The E-Prime software (28) programmed for experiments controlled stimulus presentation and reaction recording. The stimuli were displayed on a monitor with a resolution of 2880 × 1800 pixels. The CPU clock frequency of the computer is 2.6 GHz. Participants' reactions were recorded using a standard keyboard.

### 2.4 Affective flanker task

The affective flanker task assesses individuals' inhibition functions within affective contexts (29). The experimental materials consist of 40 images of facial emotions selected from the Chinese Facial Affective Picture System (CFAPS), featuring 20 male faces and 20 female faces, with an equal number of 20 positive and 20 negative emotions. A significant difference exists in valence between positive and negative faces, while no significant differences are observed in arousal, dominance, and attraction (**Table 1**). Each trial comprises five facial images, with the central target stimulus having two valence types: positive and negative. The lateral distractor stimuli also display two valences: positive and negative. The experimental stimuli encompass congruent and incongruent conditions. Under the congruent condition, the central target stimulus matches the valence of the lateral distractor stimuli, both being either positive or negative. In the incongruent condition, the central target stimulus opposes the valence of the lateral distractor stimuli. The number of trials is equal across all conditions. The entire stimulus is represented as a 2001 (width) × 469 (height) pixel image, with all stimulus images having a black background.

**Table 1:** The selected affective face images compare dimensions of valence, arousal, dominance, and attraction.

Dimensionality	Face type	M	*SD*	*p*
Valence	Positive	5.210	0.920 0.810	< 0.001
	Negative	3.530		
Arousal	Positive	5.230	1.100 1.150	0.140
	Negative	5.770		
Dominance	Positive	5.280	0.490 0.530	0.130
	Negative	5.030		
Attraction	Positive	4.720	0.660	0.120
	Negative	4.430	0.500	

**Figure 2:**
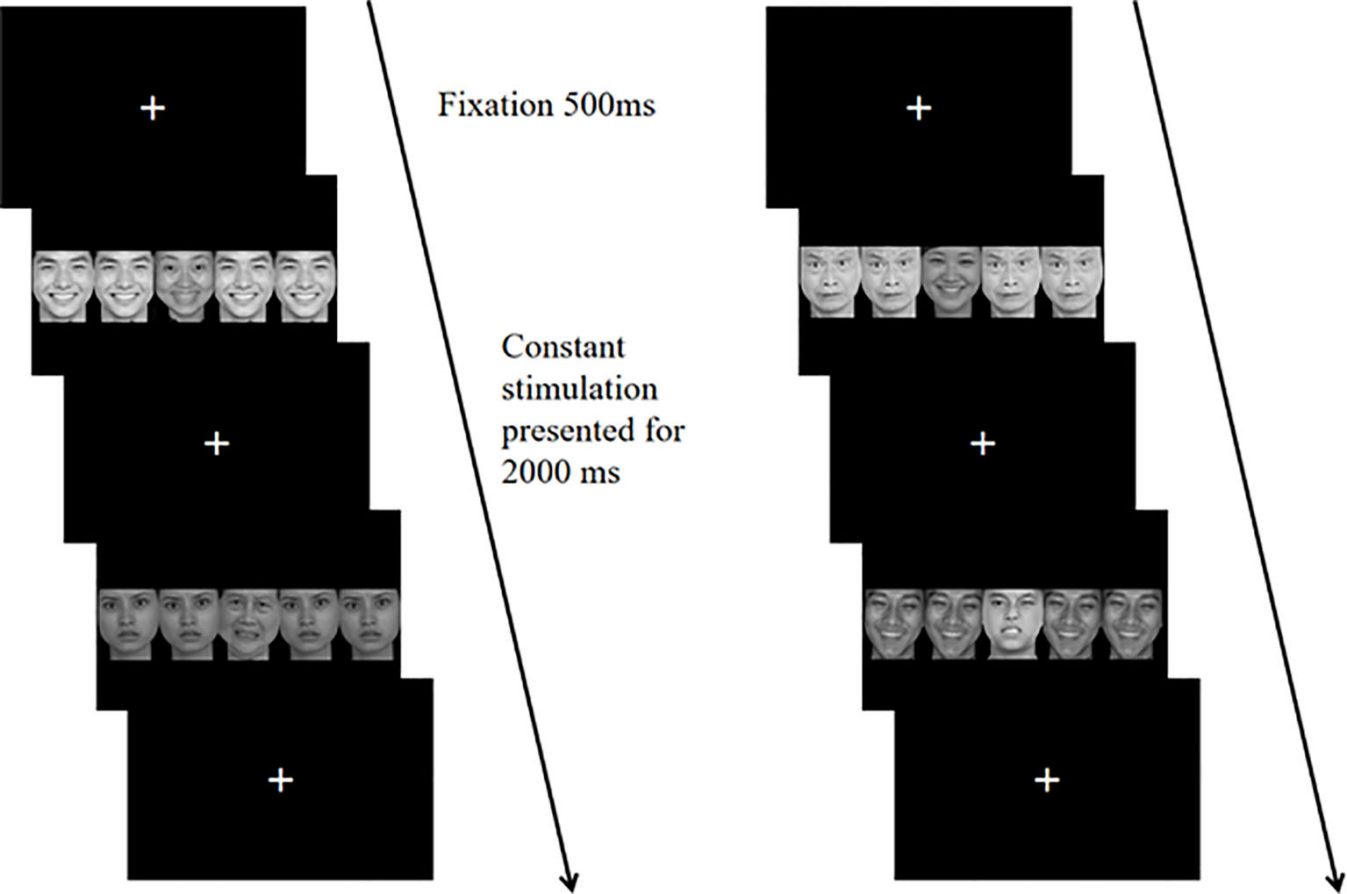
In the affective flanker task, there are two trial sequences involving affective congruent stimuli (left) and affective incongruent stimuli (right). In each sequence, the central target stimulus of the first trial is a positive emotion face, with the correct response being to press the A key. In the second trial, the central target stimulus is a negative emotion face, with the correct response being to press the L key.

The formal experiment utilizes a block design. Each block consists of 24 trials, with a total of 4 blocks. Blocks 1 and 4 involve affective congruent tasks, while blocks 2 and 3 focus on affective incongruent tasks. There is a 20-second rest interval between each block. The task requires participants to evaluate the affective valence of the central target stimuli: they press the A key for positive faces and the L key for negative faces. **Figure 2** illustrates the experimental procedure. After presenting the instructions, a fixation point appears for 500ms, followed by the experimental stimulus for 2000ms. Participants must respond within 2000ms. Any incorrect key presses or failure to respond are categorized as erroneous reactions. The stimuli remain on screen for 2000ms, during which the accuracy and reaction time of each participant are recorded. Prior to the formal experiment, a practice session is provided so that participants can thoroughly rehearse until they fully understand the task. During practice, feedback regarding key accuracy is given to participants, but no feedback is provided during the formal experiment.

### 2.5 Affective flexibility task

The affective flexibility task examines individuals' shifting abilities in affective contexts (30). The experimental materials are derived from the International Affective Pictures System (IAPS), including ten images each of negative and positive valence for individuals and groups. Considering that cultural differences across countries may influence individuals' evaluations of stimulus materials in terms of valence, arousal, and dominance, IAPS's rating parameters adopt localized evaluation results from Chinese individuals (31). Significant differences exist in valence between positive and negative images, while no significant differences occur in arousal and dominance (**Table 2**).

**Table 2:** The selected affective images compare valence, arousal, and dominance.

Dimensionality	Face type	M	*SD*	*p*
Valence	Positive	6.940	0.710 0.820	< 0.001
	Negative	3.260		
Arousal	Positive	5.190	0.870 0.540	0.310
	Negative	4.950		
Dominance	Positive	4.770	1.260 0.790	0.560
	Negative	4.570		

**Figure 3:**
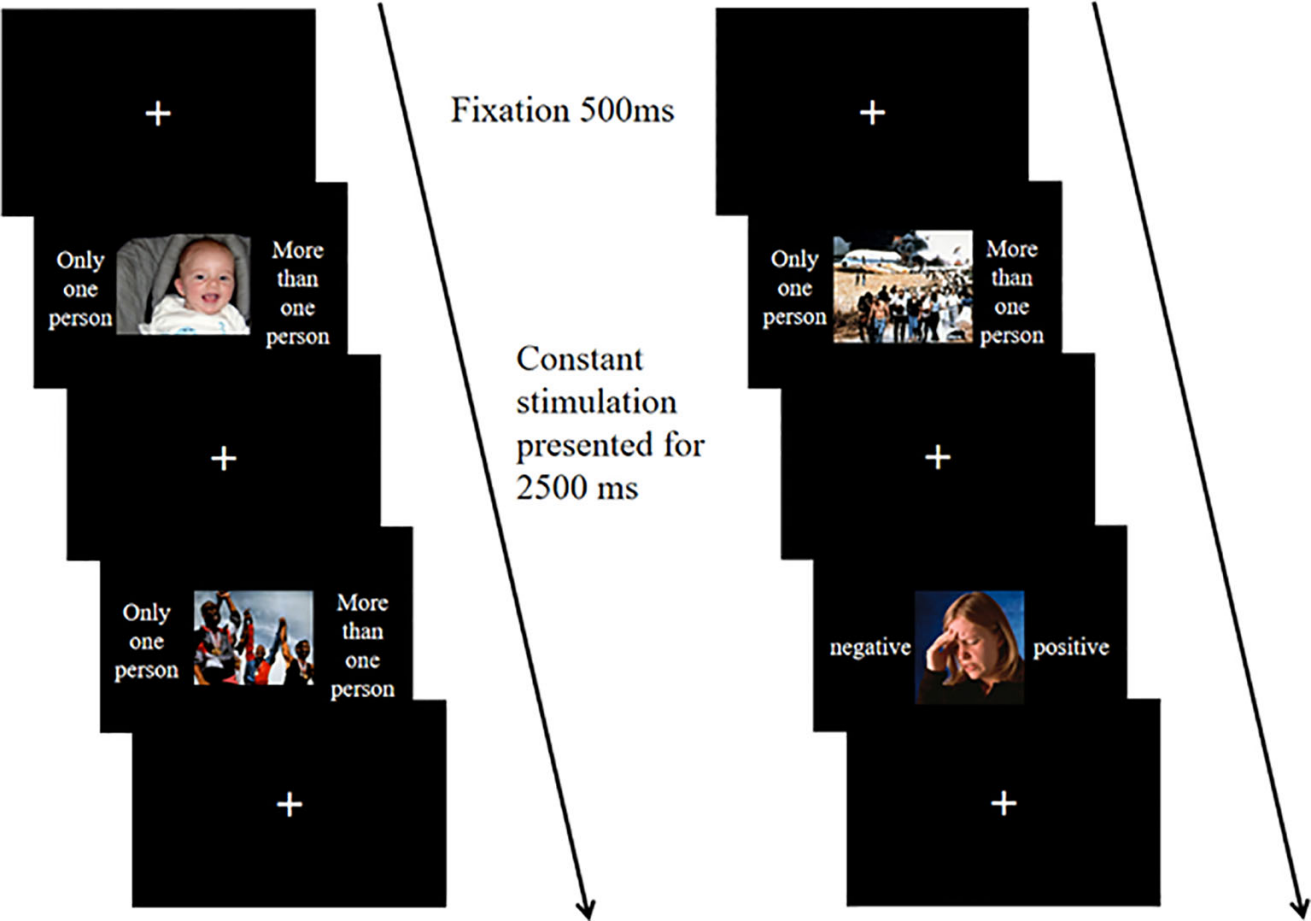
In the affective flexibility task, the repeat condition (left) and switch condition (right) consist of two trial sequences. In the repeat sequence, both trials are non-affective categorization stimuli, with the first trial pressing the A key and the second trial pressing the L key. In the switch sequence, the first trial is a non-affective categorization stimulus, where the L key is pressed, and the second trial is an affective categorization stimulus, where the A key is pressed.

The formal experiment consists of three blocks. Block 1 contains 20 trials, where participants must determine if the number of people in the image exceeds one. Only one person presses the A key, while more than one person presses the L key. Cue hints appear on both sides of the image, with non-affective hints stating "only one person" or "more than one person." Block 2 also consists of 20 trials, focusing on judging the affective valence of the images; participants press the A key for negative images and the L key for positive images. Affective cues appear on both sides of the image, labeled as "negative" or "positive." Block 3 includes 80 trials, where both types of cues will appear. Upon seeing affective cues (negative and positive), participants must assess the affective valence of the image. For non-affective cues (only one person or more than one person), they must decide if the number of people exceeds one. Cue hints appear on both sides of the images, all stimuli are presented against a black background. The task of the experiment is to respond appropriately to the different cues presented. **Figure 3** illustrates the experimental procedure. After presenting the instructions, a fixation point appears for 500ms, followed by the stimulus for 2500ms, during which participants must respond. Pressing the wrong key or failing to respond counts as an error. The stimulus lasts for 2500ms, and participants' accuracy and reaction times are recorded. Each block includes practice sessions to ensure participants fully understand the tasks, with feedback on keystroke accuracy provided during practice but not during the formal experiment.

### 2.6 Data analysis

Data analysis was conducted using SPSS 26.0. Categorical data were expressed as counts or percentages (%), and comparisons between two groups employed the *χ^2^* test. For continuous data, the Shapiro-Wilk test assessed normality. Normally distributed data were presented as mean ± standard deviation (`x ± s), with homogeneity of variance checked using Levene's test. Non-normally distributed data were represented as median and interquartile range [M (P25, P75)]. Group comparisons utilized the Kruskal-Wallis rank sum test. Correlation analysis between variables, depending on their normality, used either Pearson correlation or Spearman correlation. The Confidence Interval (CI) for all statistical analyses was set at 95%, with p < 0.050 considered significant.

We conducted a 2 (groups: GAD, HC) × 2 (stimuli: congruent, incongruent) repeated measures ANOVA on reaction times (RT) and accuracy (ACC) within the affective flanker task. We applied Bonferroni correction to the results and reported partial eta squared for power estimation. We used the flanker effect size to illustrate inhibition function; a larger value indicated poorer inhibition. We determined the RT flanker effect size as the reaction time under incongruent conditions minus the reaction time under congruent conditions. We calculated the ACC flanker effect size as the accuracy under congruent conditions minus the accuracy under incongruent conditions. We performed independent samples t-tests on the RT and ACC flanker effect sizes. In the affective flexibility task, we conducted a repeated measures ANOVA on reaction times and accuracy across 2 (groups: GAD, HC) × 2 (stimuli: non-affective categorization, affective categorization) × 2 (tasks: switch, repeat). Results were corrected using Bonferroni, and we reported partial eta squared for power estimation. The shifting function was expressed through shifting costs; higher values indicate poorer shifting function. Affective RT shifting cost = reaction time for affective switch trials - reaction time for affective repeat trials. Affective ACC shifting cost = accuracy for affective repeat trials - accuracy for affective switch trials. Non-affective RT shifting cost = reaction time for non-affective switch trials-reaction time for non-affective repeat trials. Non-affective ACC shifting cost = accuracy for non-affective repeat trials - accuracy for non-affective switch trials. We performed independent samples t-tests on affective RT shifting cost, affective ACC shifting cost, non-affective RT shifting cost, and non-affective ACC shifting cost.

## 3 Results

### 3.1 Demographic and clinical characteristics

There was no significant gender difference between the GAD patient group and the HC group, and the age difference was also insignificant. However, the difference in GAD - 7 scores between the two groups was significant, with the GAD patient group having higher scores. Similarly, the GAD patient group showed significantly higher scores on the HAMA compared to the HC group (**Table 3**).

**Table 3:** Descriptive statistics of demographic data and clinical characteristics.

Variant	GAD (*N*=50)	HC (*N*=50)	*t*/*χ²* ^a^	*p*
Age (years)	47.220 ± 5.520	49.540 ± 9.670	1.474	0.145
Gender (F/M)	14.000/36.000	14.000/36.000	0.000	1.000
GAD-7	9.600 ± 3.260	2.640 ± 2.400	12.153	< 0.001
HAMA	19.300 ± 2.690	2.200 ± 2.580	32.399	< 0.001

GAD-7, Generalized Anxiety Disorder Scale-7; HAMA, Hamilton Anxiety Scale.

a The chi-square test analyzes gender, while independent samples t-test evaluates age, GAD-7 scores, and HAMA scores.

### 3.2 Affective flanker task

#### 3.2.1 Accuracy

In the accuracy results, a significant main effect of the group was observed, with the accuracy of the HC group being greater than that of the GAD patient group. Meanwhile, the main effect of stimuli did not reach significance, and there was no significant interaction between group and stimuli (**Table 4**).

**Table 4:** Repeated measures ANOVA on the accuracy of two groups in the affective flanker task.

Variant	Congruent (%)	Incongruent (%)	*F*	*p*	*ŋ* ^2^_p_
GAD	88.780 ± 17.821	88.320 ± 19.081			
HC	94.820 ± 9.165	96.180 ± 6.049			
stimuli main effect			0.126	0.723	0.001
group main effect			7.542	0.007	0.071
stimuli × group			0.518	0.473	0.005

#### 3.2.2 Reaction times

**Figure 4:**
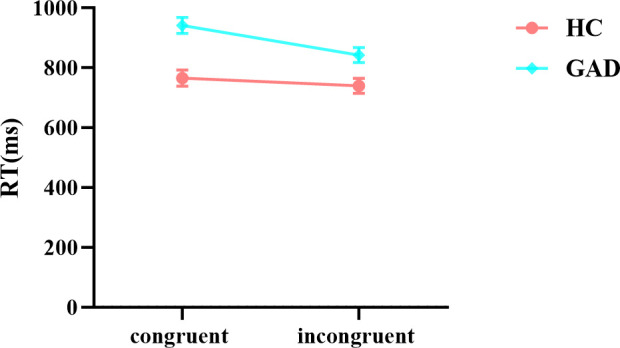
Interaction effects of reaction times between two groups in the affective flanker task.

The results of the reaction time analysis revealed a significant main effect of stimuli. The main effect of the group was also significant. Additionally, the interaction between group and stimuli was significant. Further simple effects analysis indicated (**Figure 4**) that there was no significant difference in reaction times for the HC group across different stimuli, *F* (1,98) = 2.988, *p* = 0.087, *ŋ* ^2^_p_ = 0.030. In contrast, the GAD patient group exhibited longer reaction times for congruent stimuli than for incongruent stimuli, *F* (1,98) = 44.565, *p* < 0.001, *ŋ* ^2^_p_ = 0.313 (**Table 5**).

**Table 5:** Repeated measures ANOVA of reaction times between two groups in the affective flanker task.

Variant	Congruent (ms)	Incongruent (ms)	*F*	*p*	*ŋ* ^2^_p_
GAD	941.154 ± 183.508	842.119 ± 186.596			
HC	765.272 ± 192.571	739.628 ± 163.127			
stimuli main effect			35.316	< 0.001	0.265
group main effect			15.986	< 0.001	0.140
stimuli × group			12.237	0.001	0.111

#### 3.2.3 ACC flanker effect size

**Figure 5:**
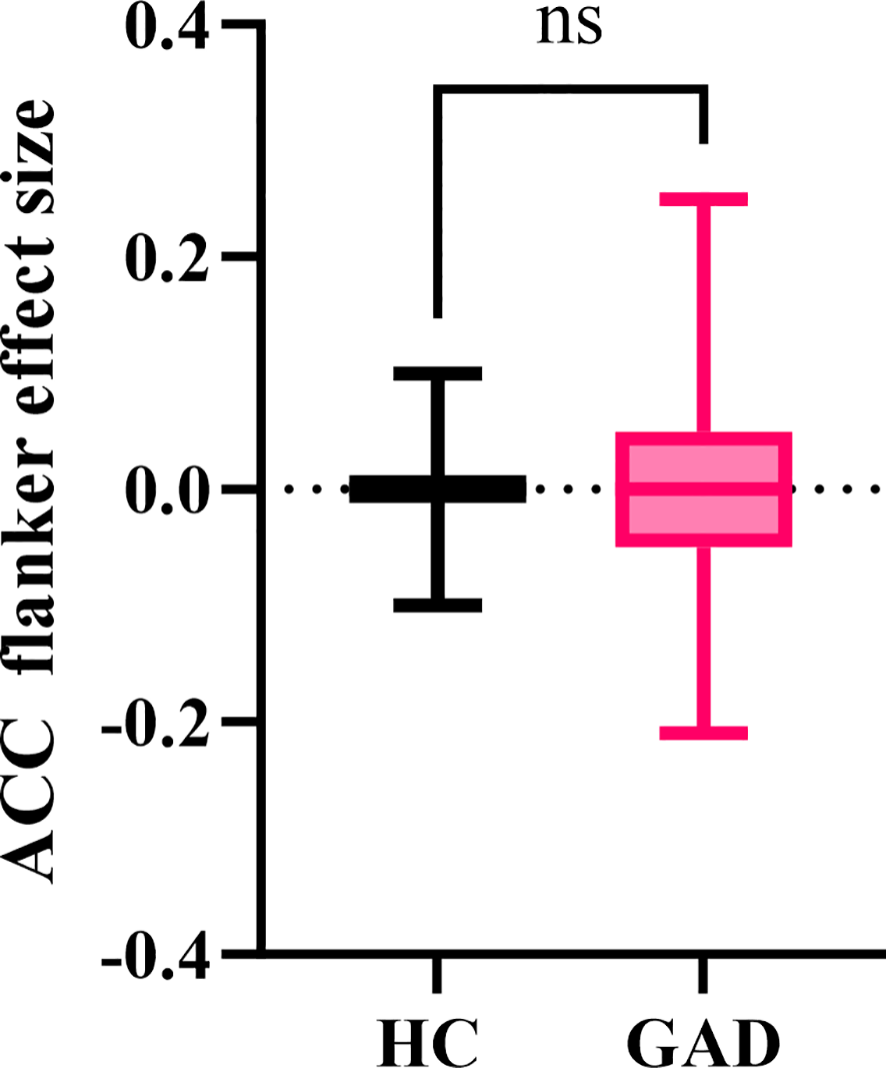
The effect size of accuracy in the affective flanker task between the two groups. ^ns^p > 0.05.

The independent samples t-test results indicated (**Figure 5**) that the GAD accuracy flanker effect size (M = -0.001, *SD* = 0.095) did not show a significant difference from the HC accuracy flanker effect size (M = -0.005, *SD* = 0.064), *t* (91) = 0.248, *p* = 0.805.

#### 3.2.4 RT flanker effect size

**Figure 6:**
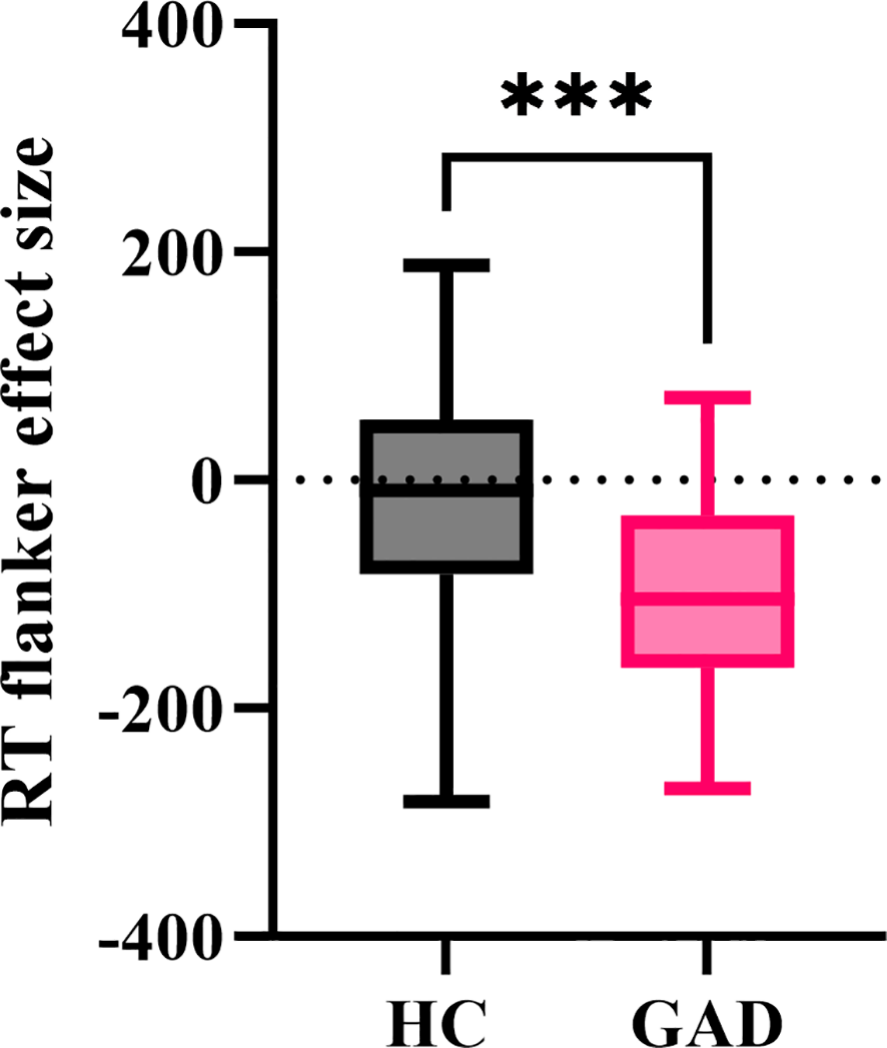
The effect size of reaction time in the affective flanker task between the two groups. ***p < 0.001.

The independent samples t-test results indicated (**Figure 6**) that the reaction time flanker effect size for GAD (*M* = -99.035, *SD* = 87.052) was significantly smaller than that for HC (*M* = -23.827, *SD* = 100.217), *t* (96) = 3.971, *p* < 0.001.

### 3.3 Affective flexibility task

#### 3.3.1 Accuracy

**Figure 7:**
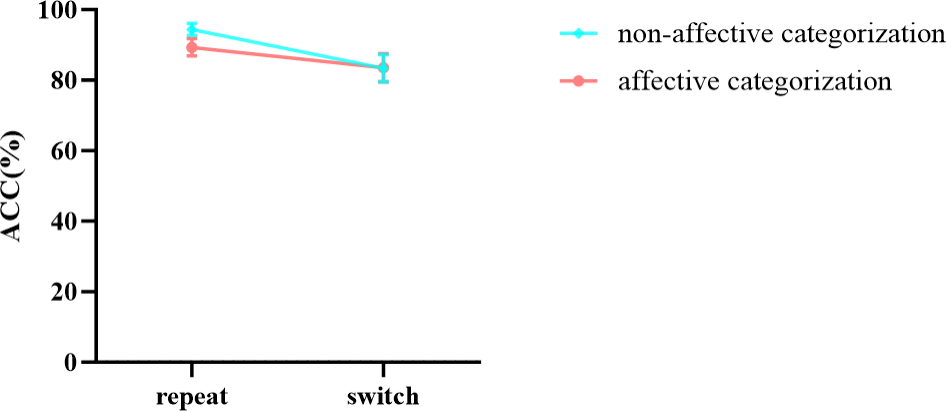
The interaction effect chart of the accuracy rates for different stimuli across two tasks.

**Figure 8:**
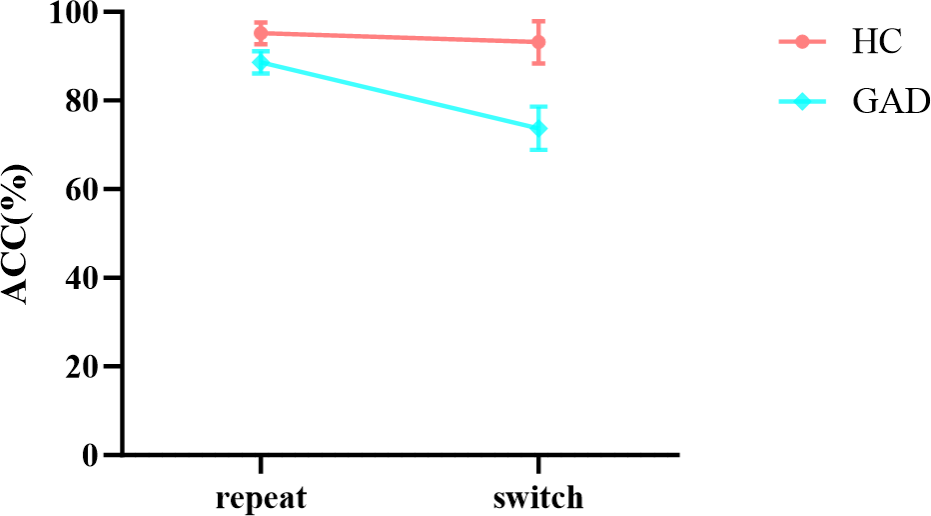
The interaction effect chart of the accuracy rates for two groups across different tasks.

In the results of accuracy (**Table 6**), the main effects of stimuli, task, and group were all significant. However, the interaction between group and stimuli was not significant, nor was the three - way interaction among stimuli, task, and group. Notably, the interaction between stimuli and task was significant. Further simple effects analysis revealed (**Figure 7**) that in the repeat task, the accuracy of non-affective categorization was significantly greater than that of affective categorization, *F* (1,96) = 18.475, *p* < 0.001, *ŋ* ^2^_p_ = 0.161. In the switch task, there was no significant difference in accuracy between non-affective categorization and affective categorization, *F* (1,96) = 0.004, *p* = 0.947, *ŋ* ^2^_p_ < 0.001. Moreover, the interaction between group and task was significant. Further simple effects analysis showed (**Figure 8**) that the HC group did not exhibit significant differences in accuracy across different tasks, *F* (1,96) = 0.963, *p* = 0.329, *ŋ* ^2^_p_ = 0.010. In contrast, the GAD patient group demonstrated significantly higher accuracy in the repeat task compared to the switch task, *F* (1,96) = 51.299, *p* < 0.001, *ŋ* ^2^_p_ = 0.340.

**Table 6:** Repeated measures ANOVA on the accuracy of two groups in the affective flexibility task.

Variant	Non-affective categorization (%)		Affective categorization (%)		*F*	*p*	*ŋ* ^2^_p_
	repeat	switch	repeat	switch			
GAD	92.920 ± 10.357	73.240 ± 26.670	84.270 ± 15.912	74.220 ± 25.719			
HC	95.900 ± 5.596	93.510 ± 7.042	94.400 ± 6.899	92.800 ± 12.129			
stimuli main effect					5.082	0.026	0.050
task main effect					33.671	< 0.001	0.260
group main effect					32.091	< 0.001	0.251
stimuli × task					4.436	0.038	0.044
stimuli × group					1.552	0.216	0.016
task × group					19.618	< 0.001	0.170
stimuli × task ×group					3.191	0.077	0.032

#### 3.3.2 Reaction times

**Figure 9:**
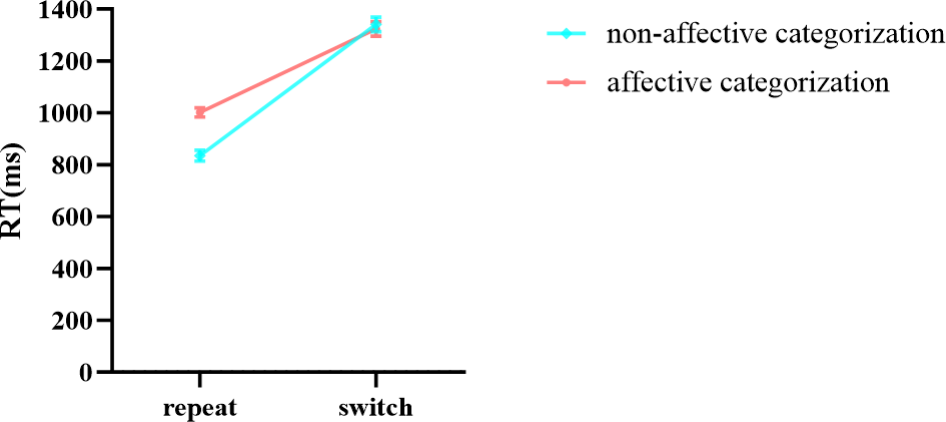
The interaction effect chart of the reaction times for different stimuli across two tasks.

**Figure 10:**
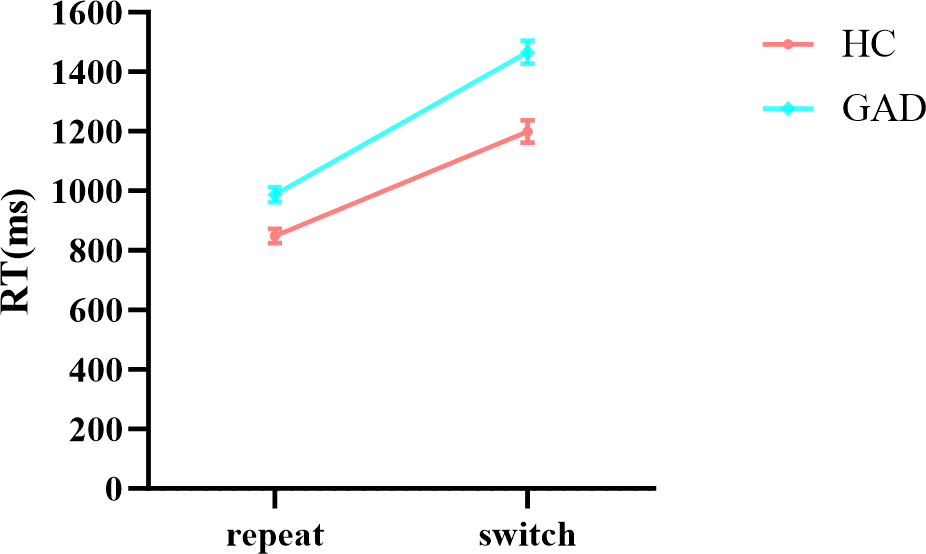
The interaction effect chart of the reaction times for two groups across different tasks.

In the results of reaction times (**Table 7**), the main effects of stimuli, task, and group were all significant. The interaction between group and stimuli was not significant, and the three-way interaction among stimuli, task, and group was not significant. Notably, the interaction between stimuli and task was significant. Further simple effects analysis showed (**Figure 9**) that in the repeat task, the reaction time for affective categorization was significantly longer than that for non-affective categorization, *F* (1,96) = 171.797, *p* < 0.001, *ŋ* ^2^_p_ = 0.642. In the switch task, there was no significant difference in reaction times between affective and non-affective categorization, *F* (1,96) = 0.833, *p* = 0.364, *ŋ* ^2^_p_ = 0.009. Moreover, the interaction between group and task was significant. Further simple effects analysis showed (**Figure 10**) that the HC group had a significantly longer reaction time in the switch task compared to the repeat task, *F* (1,96) = 119.503, *p* < 0.001, *ŋ* ^2^_p_ = 0.555. The GAD patient group also exhibited a significantly longer reaction time in the switch task compared to the repeat task, *F* (1,96) = 214.535, *p* < 0.001, *ŋ* ^2^_p_ = 0.691.

**Table 7:** Repeated measures ANOVA of reaction times between two groups in the affective flexibility task.

Variant	Non-affective categorization (ms)		Affective categorization (ms)		*F*	*p*	*ŋ* ^2^_p_
	repeat	switch	repeat	switch			
GAD	901.933 ± 225.136	1470.160 ± 316.842	1072.265 ± 178.049	1460.965 ± 341.507			
HC	767.623 ± 180.093	1210.979 ± 234.718	930.671 ± 164.334	1187.085 ± 216.096			
stimuli main effect					56.424	<0.001	0.370
task main effect					328.073	<0.001	0.774
group main effect					26.080	<0.001	0.214
stimuli × task					57.748	<0.001	0.376
stimuli × group					0.302	0.584	0.003
task × group					7.905	0.006	0.076
stimuli × task × group					0.024	0.878	<0.001

#### 3.3.3 Non-affective ACC shifting cost

**Figure 11:**
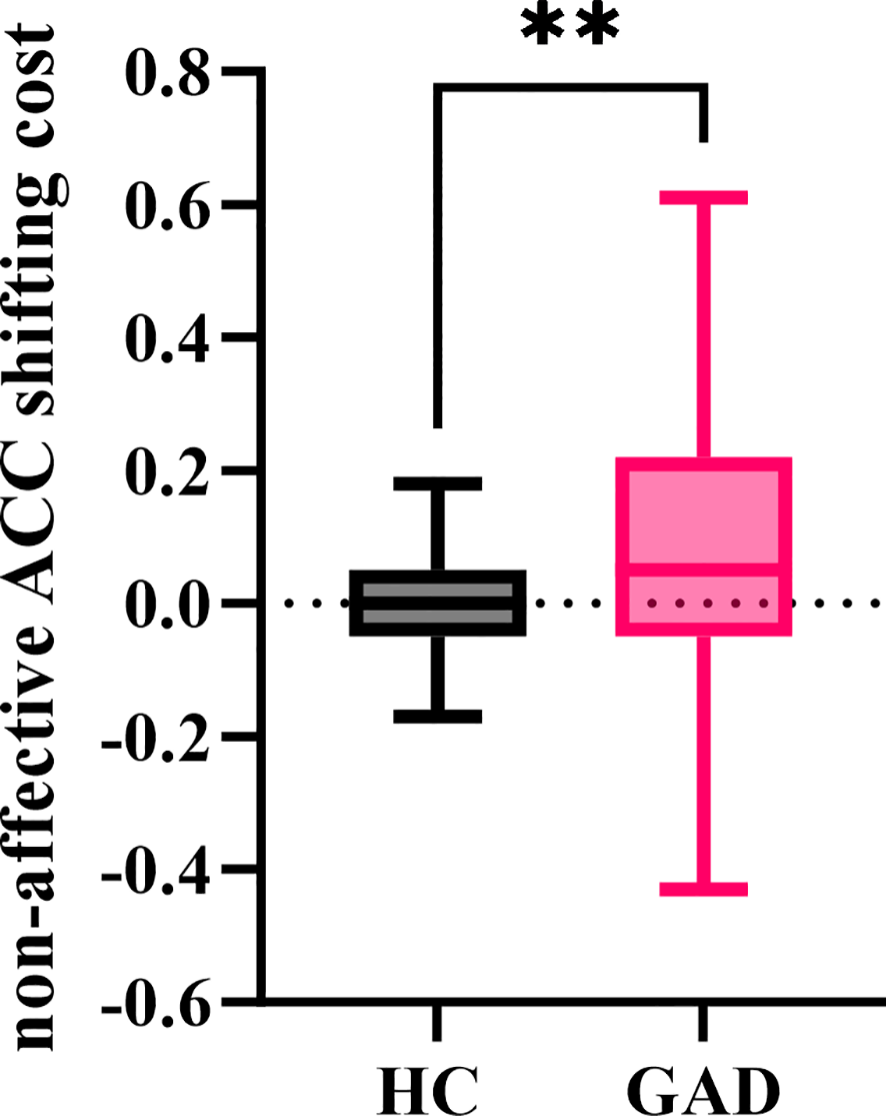
The non-affective ACC shifting cost in the affective flexibility task between the two groups. **p < 0.01.

The independent samples t-test results showed (**Figure 11**) that the non-affective accuracy shifting cost for GAD (M = 0.099, *SD* = 0.210) was significantly greater than that for HC (M = 0.009, *SD* = 0.074), *t* (95) = 2.876, *p* = 0.005.

#### 3.3.4 Non-affective RT shifting cost

**Figure 12:**
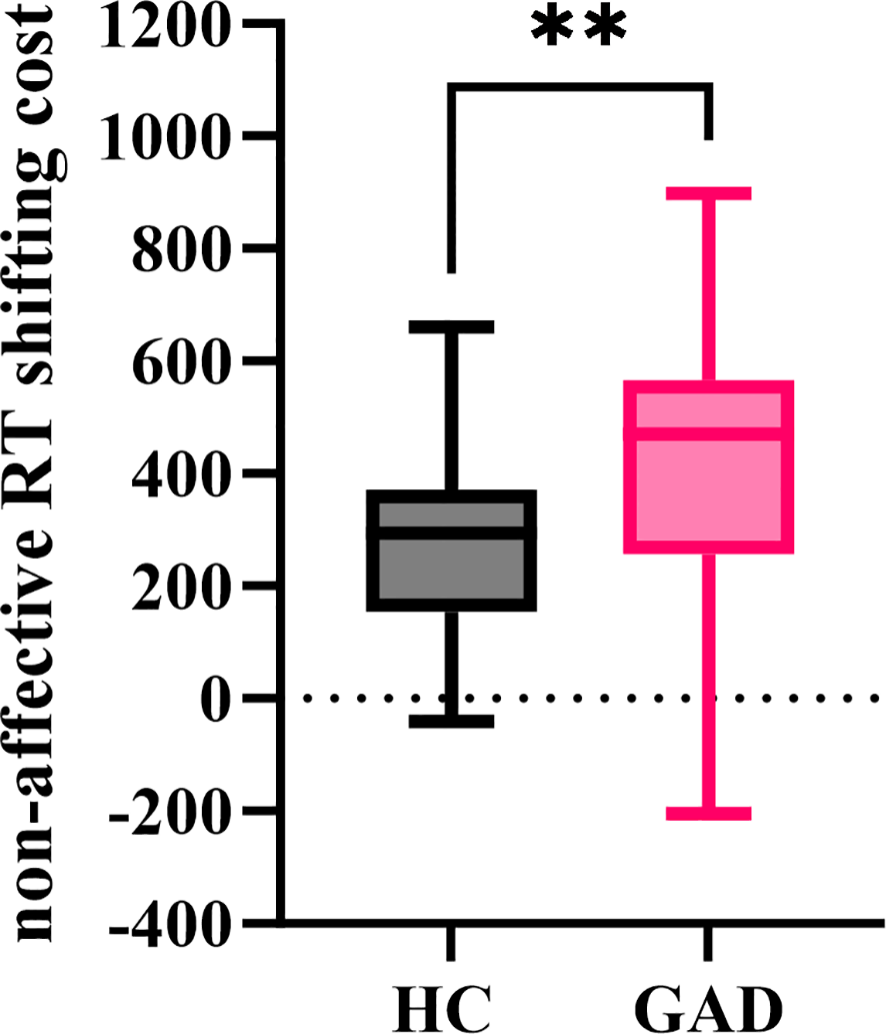
The non-affective RT shifting cost in the affective flexibility task between the two groups. **p < 0.01.

The independent samples t-test results indicated (**Figure 12**) that the non-affective RT shifting cost for GAD (M = 410.570, *SD* = 249.338) was significantly greater than that of HC (M = 268.400, *SD* = 163.456), *t* (92) = 3.295, *p* = 0.001.

#### 3.3.5 Affective ACC shifting cost

**Figure 13:**
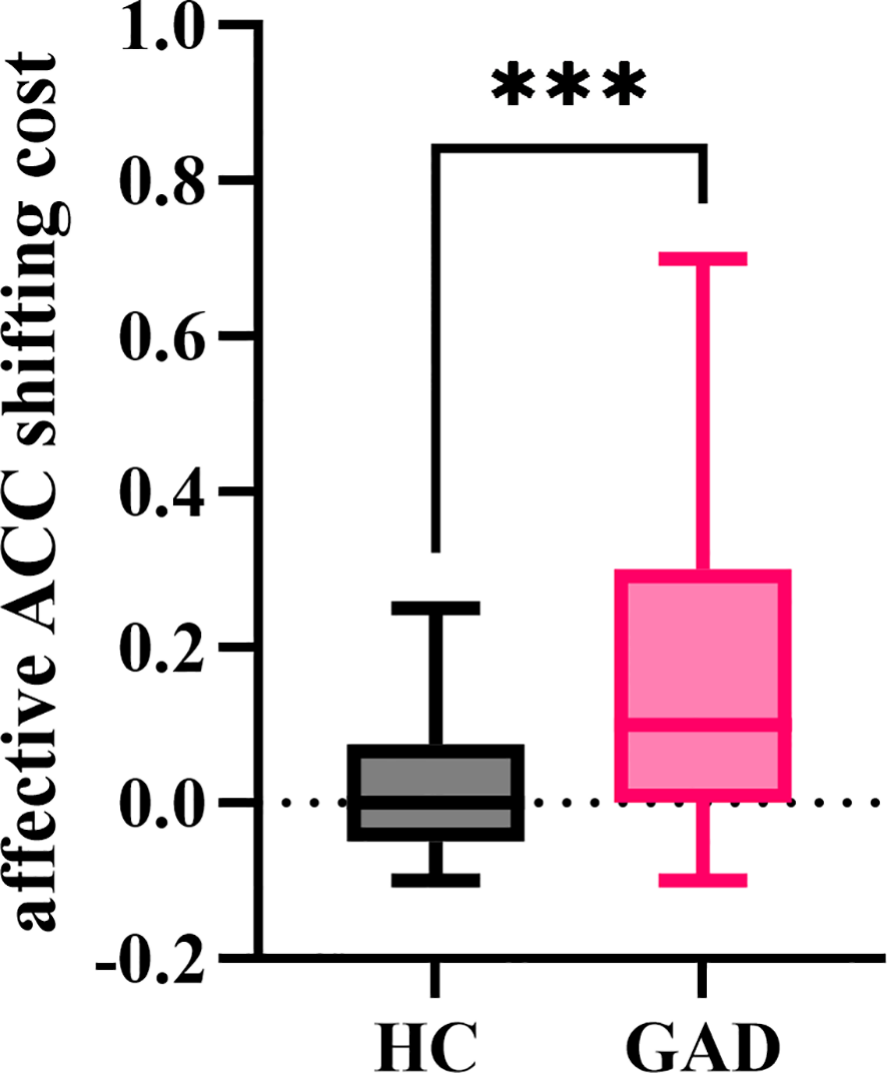
The affective ACC shifting cost in the affective flexibility task between the two groups. ***p < 0.001.

The independent samples t-test results indicated (**Figure 13**) that the affective ACC shifting cost for GAD (M = 0.171, *SD* = 0.236) was significantly greater than that of HC (M = 0.021, *SD* = 0.078), *t* (94) = 4.199, *p* < 0.001.

#### 3.3.6 Affective RT shifting cost

**Figure 14:**
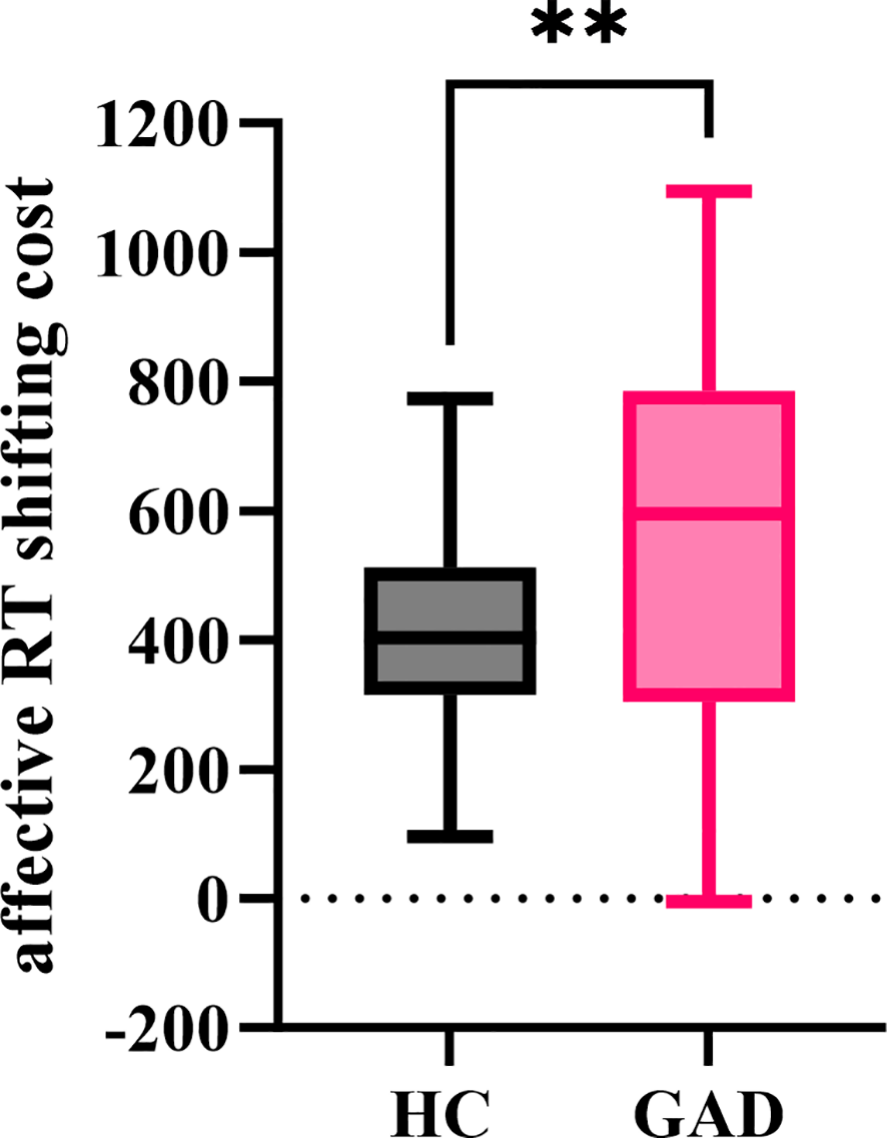
The affective RT shifting cost in the affective flexibility task between the two groups. **p < 0.01.

The independent sample t-test results indicated (**Figure 14**) that the affective RT shifting cost for GAD (M = 559.033, *SD* = 291.357) significantly exceeded that for HC (M = 413.027, *SD* = 156.076), *t* (94) = 3.060, *p* = 0.003.

### 3.4 Correlation analysis

**Figure 15:**
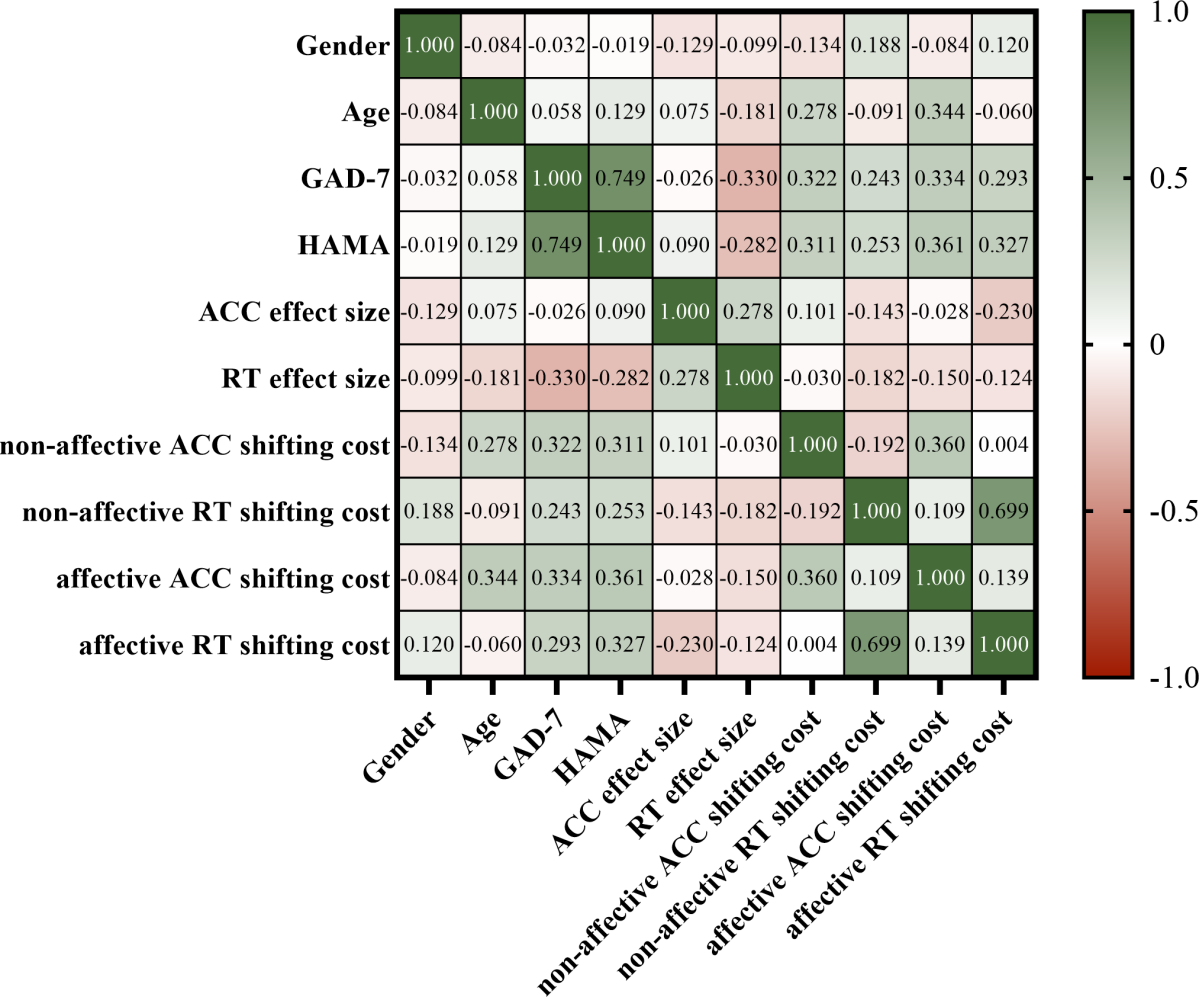
The correlation analysis between variables, with a color scale representing the variation of r values from -1.0 to 1.0.

We conducted a correlation analysis on age, gender, HAMA scores, GAD-7 scores, and behavioral performance indices from the two tasks (**Figure 15**). Age showed a significant positive correlation with the non-affective ACC shifting cost and the affective ACC shifting cost in the affective flexibility task. As age increased, the cost of accuracy transition rose. GAD-7 scores negatively correlated with the RT flanker effect size in the affective flanker task but showed significant positive correlations with the non-affective ACC shifting cost, non-affective RT shifting cost, affective ACC shifting cost, and affective RT shifting cost in the affective flexibility task. HAMA scores displayed a correlation pattern similar to that of GAD-7 scores across behavioral performance indices.

## 4 Discussion

This study examined the differences in cognitive control between GAD patients and healthy participants within affective contexts. The goal was to understand better the nature of affective control deficits in GAD patients and the underlying mechanisms of pathological anxiety. More specifically, our findings revealed that GAD patients exhibited poorer affective recognition abilities and deficits in cognitive control within affective environments. We discussed the role of these characteristics in the onset and maintenance of GAD.

The affective flanker task primarily assesses the performance of inhibition control within cognitive control under affective contexts. In this task, regardless of whether conditions are congruent or incongruent, participants achieve high levels of accuracy. However, reaction time analyses reveal that the GAD group has significantly longer response times under both conditions, indicating that GAD individuals exhibit deficits in affective recognition, and they sacrifice speed to ensure accuracy during the flanker task. This is consistent with the viewpoint pointed out in previous studies that patients with anxiety disorders exhibit slower reaction times when performing executive function tasks, while their accuracy levels are on par with those of the control group (32). This might suggest that highly anxious individuals require more cognitive resources and time for emotional regulation and inhibition control. Moreover, the research points out that the lateral frontopolar cortex (FPI) of highly anxious individuals shows hyperactivity. During affective control, they fail to engage the recruited FPI, relying instead on the dorsolateral and medial prefrontal regions. This could represent the neurobiological basis for impaired affective control in anxious individuals (33). The principal finding of the affective flanker task is that GAD patients demonstrate an enhanced detection ability under conditions which distractor stimuli conflict with target stimuli. Functionally, the Dual Mechanism of Control (DMC) can explain these results (34). The DMC posits that inhibition control encompasses two distinct processes: proactive control and reactive control. Proactive control requires anticipating and maintaining information before cognitive events, while reactive control involves detecting and resolving distractor stimuli (35). Cognitive control represents an individual’s top-down regulation to achieve established goals. Attention control theory suggests that anxiety may impair top-down cognitive control (4), prompting individuals with high anxiety to expend more cognitive resources to compensate for their performance. Given our experimental results, we propose that GAD patients show weakened proactive control related to target stimulus processing, leading to poorer performance in facial emotion recognition when compared to typical individuals. Conversely, enhanced reactive control related to distractor resolution manifests as quicker responses in incongruent conditions than in congruent ones, with an *ŋ* ^2^_p_ effect size of 0.313, which according to general standards represents a large effect. This large effect size implies that distractor stimuli have a substantial impact on the reaction times of GAD patients. This aligns with previous findings, indicating that anxiety may disrupt proactive control, possibly because anxiety consumes limited working memory capacity, thereby affecting situations requiring goal maintenance. Reactive control may be strengthened as anxiety promotes attention to threats and activates conflict monitoring systems to adjust behavior swiftly (34). Therefore, we further speculate that the abnormal inhibition control function in GAD patients during affective contexts may stem from specific mechanistic defects in their inhibition control, possibly linked to their attention allocation to threatening information and processing efficiency (36). This does not simply reflect diminished cognitive function.

Few studies have compared the cognitive control functions of GAD patients with healthy control groups, but recent reports indicate that GAD patients exhibit impairments in cognitive shifting abilities (37, 38). These findings highlight the importance of cognitive shifting and the deficits in GAD patients regarding this function. The affective flexibility task primarily investigates how shifting functions manifest in affective contexts. The ACC results from this task show a significant interaction between group and task, with simple effects analysis revealing that the HC group shows no difference in performance between repeat and switch tasks. In contrast, the GAD group performs significantly better in repeat tasks than switch tasks, with an *ŋ* ^2^_p_ effect size of 0.340, which according to general standards represents a large effect. This large effect size indicates that the type of task (repeat or switch) has a strong influence on the GAD group's ACC performance, accounting for 34% of the variance in their performance. Similar trends appear in response time results; both the HC and GAD groups require more time to switch tasks than repeat tasks, but the increase in response time is more significant for the GAD group, with an *ŋ* ^2^_p_ effect size of 0.691, which according to general standards represents a large effect. This large effect size means that the type of task has a substantial impact on the GAD group's RT performance. This indicates that switch tasks demand more cognitive resources from GAD patients. Comparative analysis of the shifting costs for GAD and HC groups shows that GAD patients have significantly higher shifting costs in both non- affective and affective tasks than the HC group. Furthermore, the difference in shifting costs during affective tasks is even more pronounced between GAD and HC groups. This is consistent with previous research, which suggests that GAD may be linked to specific affective inflexibility (39), as GAD patients manifest more significant transfer difficulties when processing affective information (40). Affective information processing impacts cognitive control, judgment, and reasoning through perceptual vigilance (which enhances information prominence) or perceptual defense (which shifts attention away from anxiety-inducing stimuli) and motivates behavior. Our study reveals that individuals with GAD exhibit reduced perceptual defense capabilities. Compared to healthy adults, they require more cognitive resources to disengage from prior tasks, indicating significant deficits in affective shifting functions. In daily situations, these deficits may result in inadequate filtering of external stimuli and information overload for GAD patients. Consequently, they may struggle to integrate this information, leading to erroneous judgments and behaviors, which in turn exacerbate their anxiety.

Previous research has focused on cognitive control training, which can enhance negative emotion management and improve affective regulation abilities (41). Some studies suggest that affective control is associated with the onset, recurrence, and maintenance of clinical mental disorders (18). Therefore, exploring the features of affective control in patients with GAD may be clinically relevant for intervening in their affective control. We hope cognitive testing can emerge as an interesting diagnostic tool: cognitive function and psychopathology are related yet independent dimensions (42). This indicates that cognitive tests might provide different types of information compared to self-reports. Although the paradigms used in this study are insufficient to replace GAD-7 as a diagnostic tool, the performance differences between GAD groups and HC groups underscore the importance of cognitive control in affective contexts within GAD-related research.

## 5 Conclusions

In sum, we found evidence that patients with GAD exhibit deficits in cognitive control within affective contexts compared to healthy adults. By measuring participants in two subcomponents of affective control (affective inhibition and affective shifting), we discovered that GAD patients show poorer emotion recognition abilities and impaired affective shifting functions. The paradigm used in this study may serve as a potential clinical diagnostic aid. However, when interpreting these findings, one must consider the limitations of the measures employed. This study did not incorporate neurophysiological indicators such as electroencephalography (EEG) or functional magnetic resonance imaging (fMRI). Future research may integrate psychological approaches with neurophysiological techniques to obtain a more comprehensive perspective and further explore the neural mechanisms of affective control.

## Data Availability

The raw data supporting the conclusions of this article will be made available by the authors, without undue reservation.
